# Novel Optimization Design Methods of Highly Loaded Compressor Cascades Considering Endwall Effect

**DOI:** 10.3390/e24060830

**Published:** 2022-06-15

**Authors:** Bo Liu, Qidong Chen, Jun Li, Xiaochen Mao

**Affiliations:** 1 School of Power and Energy, Northwestern Polytechnical University, Xi’an 710129, China; liubo704@nwpu.edu.cn (B.L.); cqd@mail.nwpu.edu.cn (Q.C.); maoxiao_chen@nwpu.edu.cn (X.M.); 2 The National Key Laboratory of Science and Technology on Aerodynamic Design and Research, Xi’an 710129, China; 3 AECC Shenyang Engine Research Institute, Shenyang 110015, China

**Keywords:** compressor cascade, optimization design, endwall effect, blade modeling methods

## Abstract

The endwall effect has a great impact on the aerodynamic performance of compressor blades. Based on three conventional near-endwall blade modeling methods of bowed blade, endbend blade and leading-edge strake blade (LESB), two combined optimization design methods of highly loaded blades have been developed considering the endwall effect in the current study, i.e., the bowed blade combined with the LESB (bowed LESB blade) and the endbend blade combined with the LESB (endbend LESB blade). Optimization designs were conducted for a compressor cascade with low solidity by using the two combined modeling methods and the three conventional modeling methods, and the optimization results were compared and analyzed in detail. The results showed that the five optimization modelling methods could all improve the performance for the original cascade, and the optimized cascade with the bowed LESB modeling method has the best aerodynamic performance. The total pressure loss of the optimal bowed LESB cascade was only 40.3% of that in the original cascade while reducing the solidity of the original cascade from 1.53 to 1.25 and keeping the static pressure rise and diffusion factor at the same level as the original one. Among the optimal cascades, the radial migration height of the low-energy fluid and the corresponding vortex have great effects on the aerodynamic performance, and the optimal bowed LESB cascade is superior to the other optimal cascades in this aspect.

## 1. Introduction

The designers of modern aero-engine compressors continuously seek to increase the stage loading and improve the aerodynamic limit on the basis of ensuring high efficiency and wide stable working range. However, achieving this goal is a time-consuming and experience-dependent process. The introduction of an optimization design method can greatly improve the compressor aerodynamic design efficiency and save design cost [1,2].

The choice of optimization design method is very important. Generally, an optimization method with stronger searching ability, combined with a reasonable amount of computing duration, can not only fulfill aerodynamic designs efficiently but also explore the compressor aerodynamic design limit to some extent. Optimization methods can be roughly divided into two categories, i.e., local optimization and global exploration. In recent years, researchers have conducted a lot of research on local optimization techniques and made a lot of progress [3,4,5,6,7,8]. When the initial search point is given, the local optimization method needs to use the gradient information at each sample point to update the next calculation position, so that it can gradually converge to an optimal value. However, when there are too many variables, we need to perform a considerable number of CFD calculations just to obtain the derivative. In order to solve this problem, the adjoint optimization approach is proposed [7,8], and it is widely applied in the field of compressor optimization design. The local optimization method is very effective for smooth and single-peak objective functions, but when it faces complex nonlinear problems, the optimal solution obtained by this method is often local rather than global.

Global exploration is another group of optimization methods, such as the genetic algorithm (GA), particle swarm optimization (PSO) and artificial bee colony algorithm (ABC). There is another new optimization method named surrogate-based optimization (SBO) [9,10,11], which is also widely used in compressor design because of its high search efficiency and fewer calculation sample points. However, still in some cases, the efficiency of SBO is not so good, and the obtained optimal point is not necessarily the real global optimal point.

The global exploratory method has a long history of development, and it has played an important role in the field of compressor aerodynamic design as an effective optimization design method [12,13,14,15,16,17,18,19]. In centrifugal compressors, due to the complex flow field, the global exploratory method is wildly used because it is difficult to directly carry out aerodynamic design. Omidi [15] accomplished an efficient and stable design of centrifugal compressors by proposing a hybrid optimization method based on GA to optimize the impellers. About flow control, Sun [16] optimized the slot structure of a compressor blade near the endwall to suppress corner separation, and non-dominated sorting genetic algorithm 2 (NSGA2) was carried out to optimize the slot to obtain the minimum total pressure loss in the cascade. Sometimes, global exploration has been used on throughflow method to optimize the compressor [16]. In some cases, global exploratory method is also used to assist the design process of a compressor, such as optimizing the hyper parameters of the other algorithms [18,19]. In general, compared to local optimization, global exploration usually requires more CFD calls to achieve its powerful exploration capacity. However, as long as a reasonable parameterization method and good exploratory algorithm are adopted, the design cycle can also be greatly shortened. In this study, a simple and efficient parameterization method and an improved artificial bee colony algorithm are used to optimize cascade.

In the design process of compressor blades, the endwall effect has a huge influence on the aerodynamic performance of compressors. As the compressor load increases, the boundary layer near the endwall grows rapidly, which can lead to significant secondary flow loss and flow blockage and bring a significant impact on aerodynamic performance [20]. So far, many flow control methods have been proposed by scholars to improve the flow field near the endwall [20,21,22,23,24,25,26,27,28]. From previous research, we can find that it is of great significance to improve the flow field near the endwall by applying bowed blade modeling methods [20,21,25,27,28] and endwall blade modeling methods [20,22,23,24,25] in the blade design of highly loaded compressors.

Although various optimization algorithms tend to be mature, the powerful global exploration ability of the global exploration method cannot be replaced. In recent years, few studies have used the global exploration algorithm to perform aerodynamic optimization design for three-dimensional (3D) blades, so relevant research is meaningful. In this paper, an optimization design platform for highly loaded cascades was developed, including five near-endwall blade modeling methods, and it is used to optimize the design of highly loaded cascades to reduce the endwall effects. The paper is organized as follows. The numerical simulation method is introduced in Section 2. Then, in Section 3, five endwall modeling methods including two combined modeling methods will be introduced in detail, and they will be used to parameterize the cascade. The optimization methods used and related information are introduced in Section 4. Then, in Section 5, the results of the optimization designs are discussed in detail. Finally, conclusions are given in Section 6.

## 2. CFD Simulation

In the present optimization study, NUMECA software was used to perform the cascade flow field simulation. Because of its high computational accuracy and efficiency, it has been widely used in the numerical simulations of turbomachinery.

All computation grids were generated by AutoGrid5 module in NUMECA. In order to ensure the high quality of grids, a block grid structure (also called O4H gird) was adopted, and the grid was refined at the near-endwall of the cascade. The steady flow model was chosen, and flow simulation was set up as follows. The Spalart–Allmaras (S–A) turbulence model, a one equation model, was adopted. The inlet boundary had been set to an evenly distributed condition, inlet total temperature was 288.15 K and inlet total pressure was 101,325 Pa. On account of the inlet Mach number and the cascade’s inlet flow angle should be fixed in the process of optimization, the outlet boundary was given a fixed outlet mass flow rate and initial pressure. Adiabatic and non-slipping condition were set on the walls. EURANUS solver was utilized, which uses steady time-advancing method to solve Reynolds time-averaged Navier–Stokes equations.

**Figure 1:**
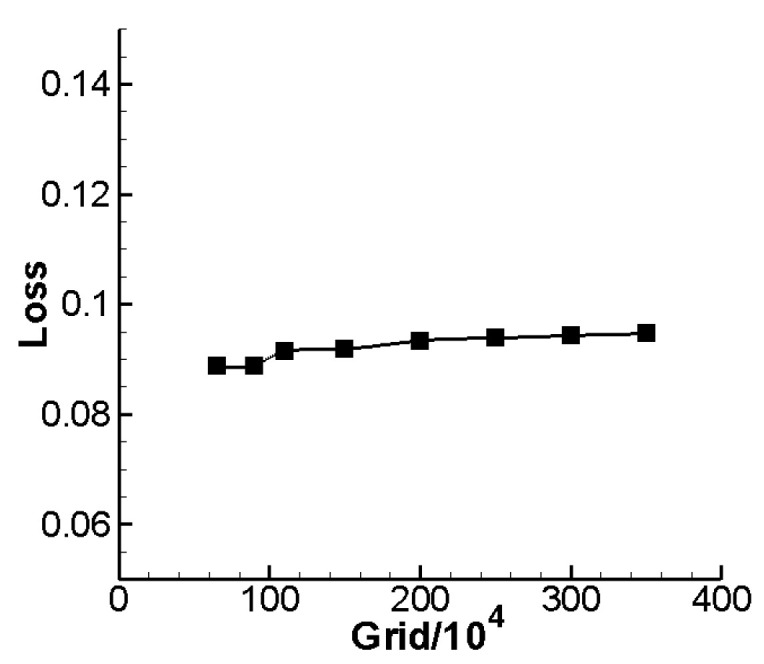
Total pressure loss in different number of grid nodes.

**Figure 2:**
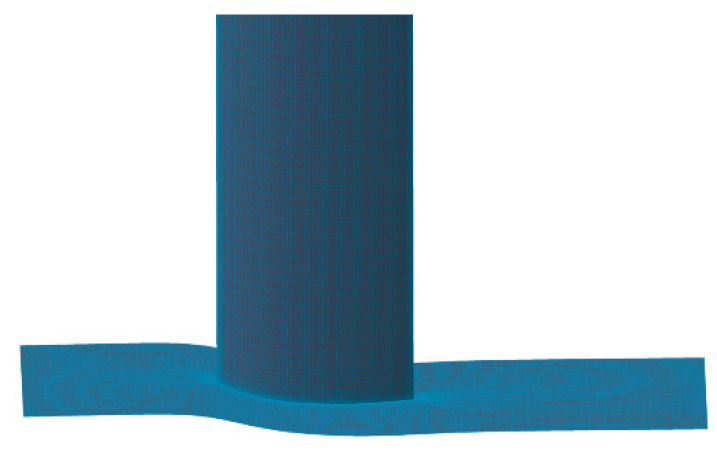
Cascade calculation mesh.

Grid dependency study was performed, where 0.65, 0.9, 1.1, 1.5, 2.0, 2.5, 3.0 and 3.5 million grid nodes were calculated under design condition (0° angle of attack, 0.7 inlet Mach number). Figure 1 gives the comparison figure of total pressure loss under diverse grid nodes number. From Figure 1, we can find that when the grid size gradually become smaller, total pressure loss gradually increases, and when the number of grid nodes reaches 1.1 million, the variation range of loss will be significantly reduced. On the basis of compute accuracy, grid number should be as low as possible to save the time of optimization design. So, all in consideration, 1.1 million was thought to be suitable and will be used throughout this study. Moreover, in order to clear off the influence of endwall mesh scale, Table 1 gives the contrast calculation of different endwall mesh scales under the design condition. From Table 1, we can find that when the endwall mesh scale reaches $5\times 10^{-6}$ m, the result of flow calculation almost keeps still, so $5\times 10^{-6}$ m is chosen as the endwall mesh scale throughout optimization numerical calculation, and m is chosen as the endwall mesh scale throughout optimization numerical calculation, and y+ value was less than 3 near the wall under this condition. The cascade grid used for the calculation is given in Figure 2. In order to guarantee the consistency and accuracy in the process of design optimization, the mesh topology structure will stay unchanged.

## 3. Cascade Endwall Modeling Methods

In order to promote the aerodynamic performance at the endwall area, we need to modify blade shape and blade stack law near the endwall. The original cascade in this study is a compressor stator cascade, and we call this original cascade a straight cascade, which will be modified to improve its aerodynamic performance. This section will introduce five different kinds of endwall modeling methods, respectively, i.e., bowed blade, endbend blade, leading-edge strake blade (LESB) blade, bowed blade combined with LESB blade (bowed LESB blade) and endbend blade combined with LESB blade (endbend LESB blade). These modeling methods will be introduced in detail.

### 3.1. Bowed and Endbend Blade Modeling Method

**Figure 3:**
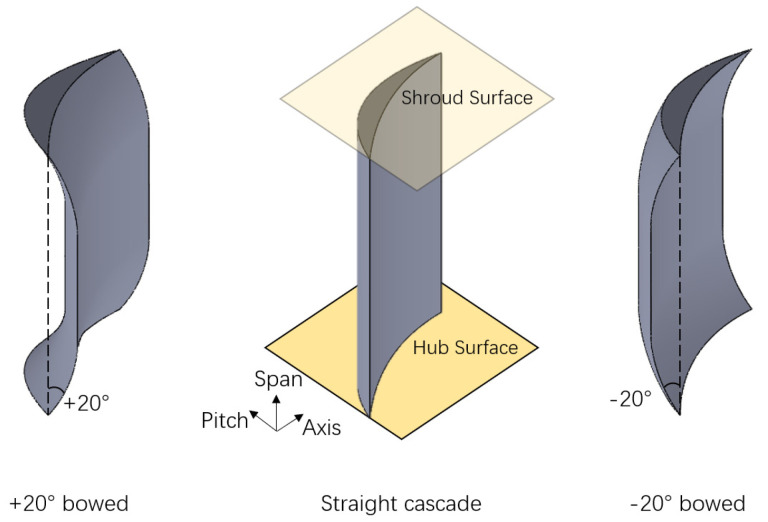
Schematic of bowed blade.

**Figure 4:**
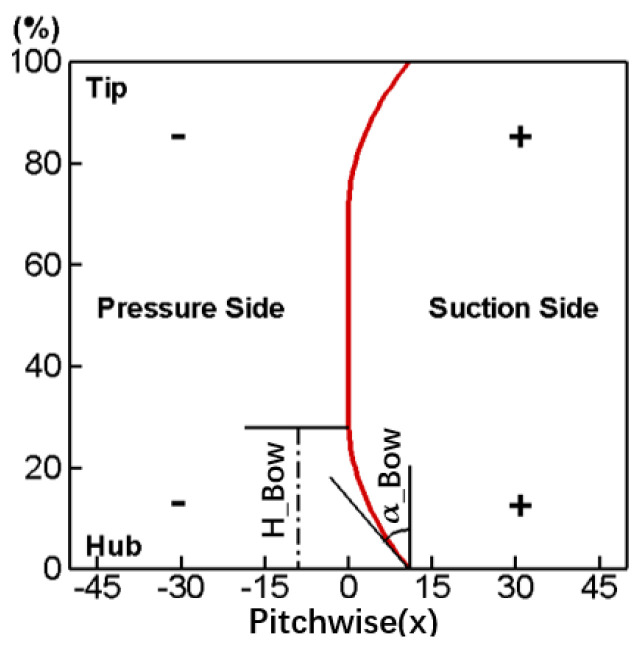
Stack line of bowed blade.

Compared to a straight cascade, a bowed blade means changing the cascade stack line along the pitch-wise direction as shown in Figure 3. In addition, Figure 4 gives the variation rule of a stacking line in a bowed blade. It divides the cascade stack line into three sections, and from hub to shroud are the parabola part, the straight part and the parabola part, respectively. The variate H_Bow in the figure represents the height of the parabola part along the spanwise direction, and the variate α_Bow represents the bowed angle at the endwall. The exact form of the parabolic equation can be determined after giving H_Bow and α_Bow. Regarding the sign of the variable, the movement of the stacking line towards the suction side and towards the pressure side are defined as the positive bow and the negative bow, respectively, which can also be seen in Figure 3.

**Figure 5:**
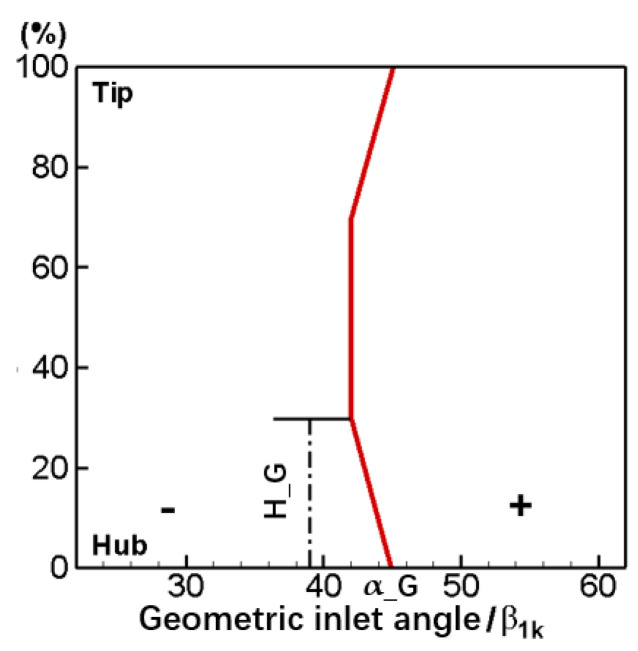
Geometric inlet angle distribution of endbend blade.

The endbend blade completes the endwall modeling by adjusting the geometric inlet angle or stagger angle of the blade near the endwall, and this study chooses to adjust the geometric inlet angle. Figure 5 gives the schematic of the geometric inlet angle distribution of the endbend blade. The variate α_G represents the maximal endbend angle (relative to the geometric inlet angle of straight blade), and the variate H_G represents the height of geometric inlet angle adjustment along the spanwise direction. In addition, we stipulate it as the positive endbend when the geometric inlet angle is bigger than the straight blade and vice versa. The adjustment rule of the geometric inlet angle near the endwall is stipulated to be linear, as shown in the Figure 5. When raised to height H_G, the geometric inlet angle will remain a fixed value equal to the straight cascade. In the modeling process, when changing the geometric inlet angle of the blade, we need to ensure that the geometric outlet angle, blade chord length, blade maximum thickness, relative position of blade maximum thickness, the distribution of camber, etc., all remain unchanged.

### 3.2. LESB Balde Modeling Method

**Figure 6:**
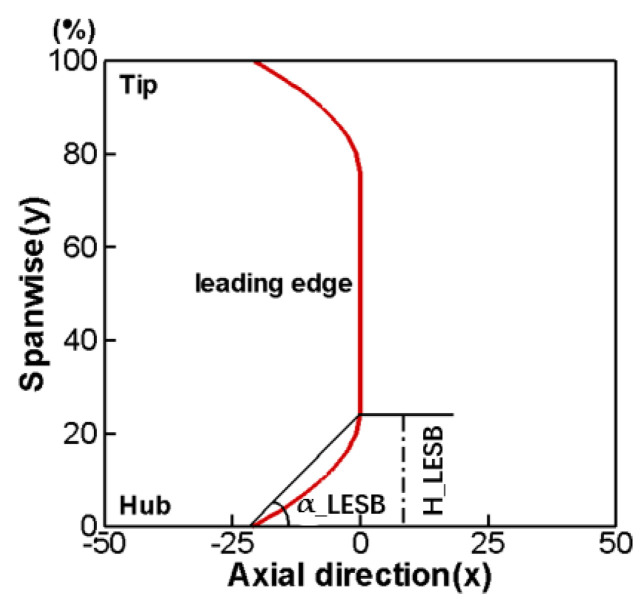
Distribution curve of axial chord length variation of LESB blade.

The LESB blade first originated from the strake wing in the aircraft airfoil, which can postpone airfoil stall at a high angle of attack. Applying the idea of the strake wing on compressor blades can effectively improve the flow conditions at the endwall area and enhance the aerodynamic performance of the cascade [29]. In the process of blade modeling, specifying the axial chord length distribution of the LESB blade is very important, and this study selects parabolic law to describe the distribution. Figure 6 shows the distribution curve of the axial chord length variation of the LESB blade along the spanwise direction, and the curve can be divided into three sections: the parabola part on both sides near the wall and the straight line in the middle. The variate H_LESB means the height of the LESB blade along the spanwise direction, and the variate α_LESB means the angle between the axial and the parabolic part of the LESB blade.

**Figure 7:**
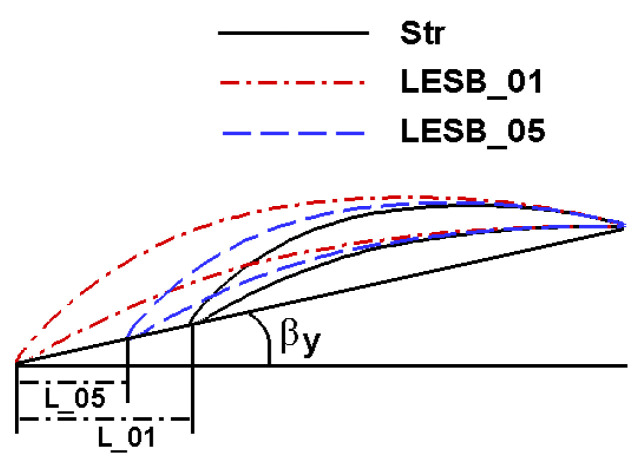
New modeling process of LESB blade.

After specifying the axial chord length of the blade profile at a different span position, how to trim the shape of the airfoil according to that chord length is also a problem. A new LESB blade modeling method that is different from the traditional modeling method is proposed. Figure 7 shows the new modeling process. Str in the figure legend represents the straight blade airfoil; LESB_01 in the legend represents the first cross section of n cross sections along the spanwise direction, which is also the nearest section to the endwall; LESB_05 in the legend represents the fifth cross section of n cross sections along the spanwise direction; L_01 and L_05 mean the increment of the axial chord length of the blade profile on the first and fifth section, respectively, and $\beta_{y}$ represents the stagger angle of the blade.

In the traditional modeling process of an LESB blade, after ascertaining the increment of chord length, two straight lines are used to smoothly connect the blade leading edge. This connection method is simple, but it is difficult to always ensure the connection with the blade leading edge is smooth, especially when the airfoil bends too much, or the pressure side curve is too steep. However, the proposed new method does not simply use straight lines to connect the blade profile but completes the modeling of the LESB and the airfoil in a unified manner to ensure the continuity of the airfoil curves. The new modeling process can be seen in Figure 7, after ascertaining the increment of the axial chord length through the distribution in Figure 6, and the real chord length on every cross section is able to be calculated by the stagger angle. Next, keeping the geometric inlet/outlet angle, maximum thickness, maximum thickness position and curvature distribution unchanged, regenerate the new airfoil on each section to generate the final LESB cascade.

### 3.3. Bowed and Endbend LESB Blade

The above three blade modeling methods are all able to improve the flow condition in the endwall region of the high-loaded cascade. Therefore, this study conceives that using a combination of two different modeling methods may have a more positive impact. There are two ways of combining; one is the bowed LESB cascade, and the other is the endbend LESB cascade. Take the bowed LESB cascade for an example. As stated in the previous chapter, H_Bow means the bowed height in the bowed blade, and H_LESB means the height of the LESB in the LESB blade. So, there are three situations when combining two modeling methods, i.e., H_Bow > H_LESB, H_Bow < H_LESB and H_Bow = H_LESB. In different cases, different spanwise ranges of blade will have different modeling methods in order to work. Some ranges only need one modeling method to work, while other ranges require both bowed blade and LESB blade modeling methods to work at the same time. In this case, when these two modeling methods are required to act at the same time, the modeling order is not important because the bowed blade only affects the blade stacking line, and the LESB blade only changes the shape of the blade section. However, things are different when it comes to the endbend LESB cascade. When two modeling methods (endbend blade modeling method and LESB blade modeling method) are required to act at the same time at a certain spanwise location, the modeling order is important because both of the modeling methods will affect the blade section. In order to ensure the rationality of the modeling process, the endbend blade modeling was completed first, followed by the LESB blade modeling.

## 4. Optimization Design Strategy

The straight cascade studied in this paper is a compressor stator cascade, and the main target design parameters are given in Table 2. The original straight cascade has the same parameters as Table 2 except solidity. The initial solidity of the original straight cascade is 1.53 with a diffusion factor of 0.55. To explore the application potential of the optimization method in the high thrust/weight ratio of an aircraft engine with less compressor blades, the cascade solidity is reduced to 1.25 from 1.53. Nevertheless, reducing the blade solidity will cause higher load and hence bring larger loss due to the secondary flow near the endwall. This paper will study the full 3D optimization design of the low-solidity and highly loaded cascade with the consideration of the endwall effect, aiming to design the cascade with the best aerodynamic performance under this load condition.

**Figure 8:**
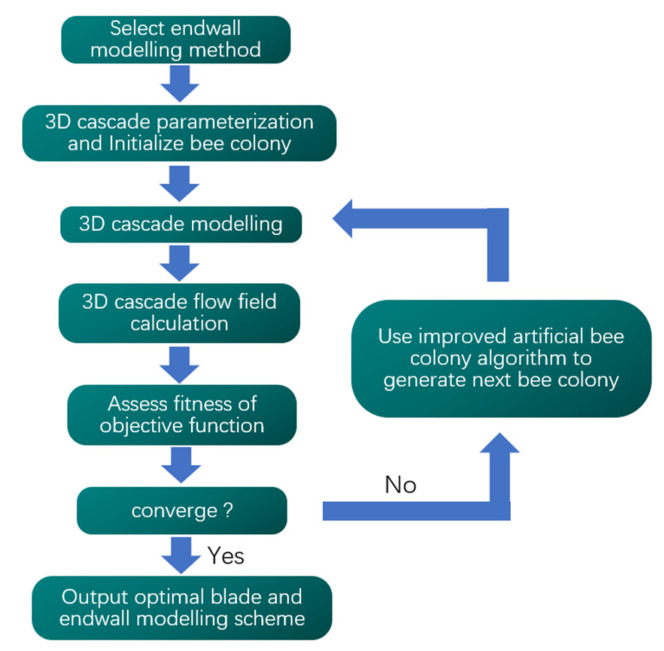
Flow diagram of cascade optimization design method considering endwall effect.

The preceding part of this paper introduced five near-endwall blade modeling methods, and this study combined these methods within an optimization design platform for highly loaded cascades. Figure 8 gives the flow diagram of the optimization design method, which can automatically and with high efficiency accomplish the highly loaded cascade optimization design.

The optimization design method first selects the blade modeling method at the near-endwall region, including the five methods described in the previous chapter. Next, it needs to parameterize the cascade and initialize the bee colony algorithm. It mainly includes two parts to parameterize the cascade: the two-dimensional (2D) airfoil on the spanwise section and the blade near the endwall. In the process of optimizing the highly loaded cascade, this study not only optimizes the endwall area but also optimizes the 2D airfoil simultaneously, so it can better match the flow field near the endwall and effectively decrease the loss of cascade. The 2D airfoil parameterization method was introduced in detail in a former study [30], namely the CLSTD (camber line superposing thickness distribution) method. The parameters such as geometric inlet angle, geometric outlet angle and maximum thickness are set to be unchanged, and CLSTD is able to use only three variates to effectively change the distribution of the loads in the front and rear parts of the airfoil. The three variates are the blade front chord ratio (BFB), blade front bend ratio (FS) and relative position of maximum thickness (XLMB). After adding an extra parameter of leading-edge radius ($R_{1}$) of the airfoil, a total of four parameters are used to change the shape of the 2D airfoil.

As described in the previous chapter, there are five different methods of endwall modeling. Table 3 lists the specific parameters of each method, wherein H_Bow is the height of the bow along the spanwise direction, H_G is the height of the endbend along the spanwise direction, H_LESB is the height of LESB along the spanwise direction, α_Bow is the bow angle at endwall, α_G is the maximal endbend angle and α_LESB is the angle between the axial and the parabolic part of LESB at the leading edge of the blade.

In order to ensure that the generated blades have considerable structural strength and avoid generating unreasonable blade shapes, it is necessary to limit the disturbance range of parameters during the optimization process. Through previous design experience and some attempts, the constraint range of parameters is set in Table 4.

After the 3D cascade parameterization is completed, the program enters the optimization algorithm module. An improved artificial bee colony (IABC) algorithm is used as the optimization algorithm because of its good performance [30,31]. Table 5 gives some parameter settings of the IABC algorithm in the optimization process.

The construction of the optimization objective function also plays an important role in the optimization design. During the optimization design process in this study, the aerodynamic characteristics of the highly loaded cascade are fully considered, and the total loss obtained by averaging the mass flow is taken as the main optimization objective. At the same time, the load is limited, that is, the diffusion factor and static pressure rise of the optimized cascade cannot be smaller than the straight cascade. Formula (1) gives the optimization objective function used in the optimization design, where the subscript lim denotes the value in the straight cascade. As shown in Formula (1), it introduces a penalty function about the diffusion factor and static pressure ratio into the optimization objective function; once either of them is lower than the value in the straight cascade, fitness will be set to a maximal value. This ensures the optimized cascade losses are minimized while the aerodynamic loads are kept at the corresponding level.

(1) $$\begin{array}{l} if\;\{\begin{array}{c} DF\geq DF_{\lim}\\ \frac{P_{2}}{P_{1}}\geq {\left(\frac{P_{2}}{P_{1}}\right)}_{\lim} \end{array}\;\;\;\;fitness=Loss\\ else\;fitness=Loss_{\max} \end{array}$$

In Formula (1):

Loss—cascade total pressure loss factor.

${\mathrm{Loss}}_{\max}$—a maximum value of Loss, set to 1.0.

DF—cascade diffusion factor, defined in Formula (2).

$\frac{P_{2}}{P_{1}}$—cascade static pressure ratio.

(2) $$DF=1-\frac{c_{2}}{c_{1}}+\frac{\Delta c_{u}}{2c_{1}\tau }$$

In Formula (2):

$c_{1}$—cascade inlet absolute velocity.

$c_{2}$—cascade outlet absolute velocity.

$\Delta c_{u}$—difference between the pitch-wise component of cascade inlet and outlet absolute velocity.

τ—cascade solidity.

## 5. Results and Discussions

### 5.1. Overall Optimization Results

Using the straight cascade as the initial design cascade for optimization and using five different near-endwall blade modeling methods to shape the highly loaded cascade, respectively, we ultimately obtained five cascades with optimal aerodynamic performance.

Table 6 lists all the values in optimization variables after the optimization design. Comparing the optimization variables of these optimal cascades, we can see that the endbend cascade, the LESB cascade, the bowed LESB cascade and the endbend LESB cascade have similar variable ranges, which are quite different from that of the bowed cascade and straight cascade.

Table 7 lists the optimization design results of the highly loaded cascade. As can be seen in the table, the aerodynamic performance obviously improved after optimization. Comparing five near-endwall modeling results, the bowed LESB cascade has the best aerodynamic performance with high aerodynamic load. Its total pressure loss is only 0.0389, and its static pressure ratio and diffusion factor are close to 1.2 and 0.6, which are all optimal levels among the five modeling methods. Among the five endwall modeling methods, the two combined modeling methods raised in this study (bowed LESB blade and endbend LESB blade) are obviously superior to the other three traditional modeling methods. Among the three traditional modeling methods, the LESB blade modeling method is the best one.

### 5.2. Optimization Results of the Cascade Flow Field

**Figure 9:**
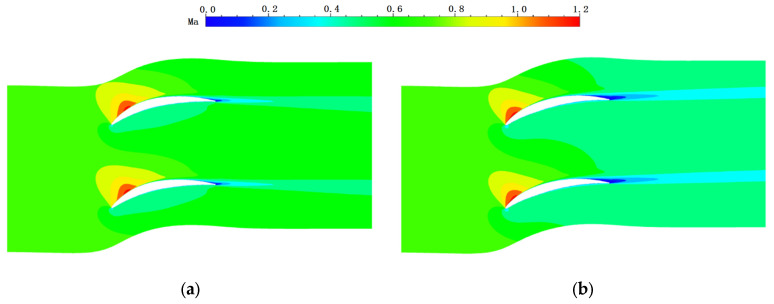
Mach number contour at 50% blade span position. (**a**) Straight cascade. (**b**) Bowed LESB cascade.

Figure 9 shows the Mach number distribution contours at the 50% span position of the straight cascade and bowed LESB cascade (the other four near-endwall modeling methods have a similar flow field as that of the bowed LESB cascade). In the figure, the flow conditions of the optimized cascades all become deteriorated to a certain extent.

**Figure 10:**
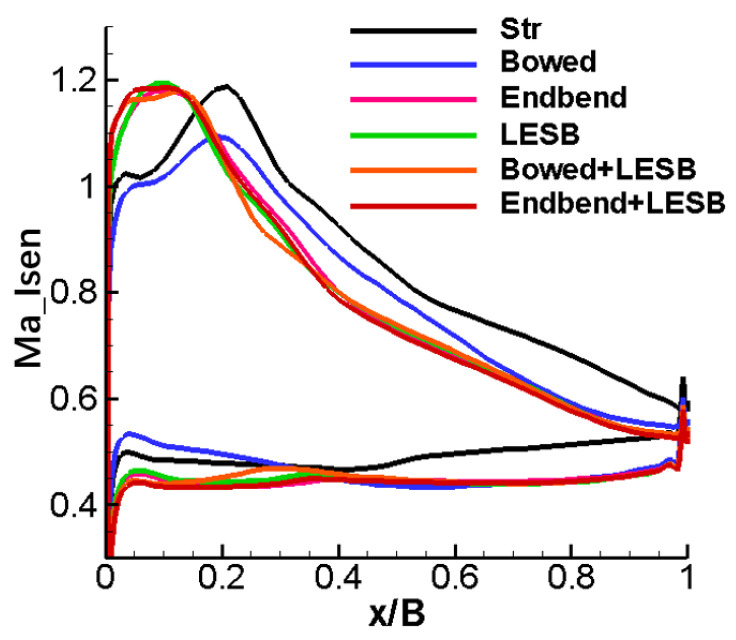
Isentropic Mach number distribution of blade surface at 50% span position.

Figure 10 shows the isentropic Mach number distribution on the blade surface at the 50% blade span position, and a different peak Mach number position and Mach number magnitude can be visualized. As can be seen in Figure 10, all the modelling cascades, except the bowed cascade, have an obvious greater load than the straight cascade before the first 20% relative chord length, less load at 20–50% relative chord length and basically the same load after 50% relative chord length. However, compared to the straight cascade, the bowed cascade has a smaller load before the first 50% relative chord length and basically maintains the same load after the 50% relative chord length.

In summary, compared to the straight cascade, the five optimal endwall modeling cascades all deteriorate their flow field in the mid-span position to a certain extent, and the mid-span load on the blade will be slightly reduced. It is the optimal bowed cascade that has the relatively most significant impact on the flow in the middle section.

**Figure 11:**
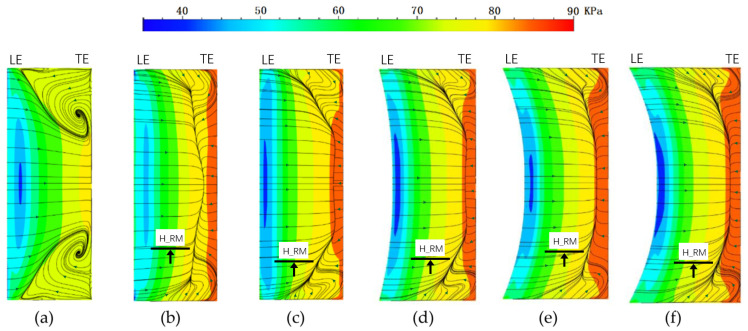
Static pressure and wall limit streamline on cascade suction side: (**a**) straight cascade; (**b**) bowed cascade; (**c**) endbend cascade; (**d**) LESB cascade; (**e**) bowed LESB cascade; (**f**) endbend LESB cascade.

Figure 11 shows static pressure and wall limit streamline on the cascade suction side. In the figure, LE means the leading edge of the cascade and TE means the trailing edge of the cascade. We also label the height of the radial migration of the endwall boundary layer as H_RM, which will be discussed in detail later. We can find in the Figure 11 that the strong adverse pressure gradient in the straight cascade causes a major reverse flow zone on the suction side, which also leads to an intense boundary layer separation. After conducting endwall modeling on the straight cascade, the five optimal cascades have a larger separation range but a simpler separation structure, so the total pressure loss of the optimal cascades is significantly smaller than the original cascade.

The position of the separation line in the five optimal cascades is different. The axial position of the separation line in the bowed cascade is more forward than the other four, that is to say, the separation region on the suction side in the optimal bowed cascade is bigger than the others. The other four optimal cascades have basically the same of axial position of the separation line. Comparing the radial initial position of the separation line, the endbend cascade is closest to the endwall, followed by the LESB cascade. The bowed LESB cascade has the closest radial initial position of the separation line, which means it has the smallest separation effect region along the spanwise direction.

A higher outlet static pressure can be observed in the five optimal cascades than in the straight cascade. Furthermore, the high static pressure areas of the cascades using two combined modeling methods are obviously bigger than that of the other three traditional modeling methods. In other words, a stronger pressure diffusion ability can be obtained by using two combined modeling methods.

**Figure 12:**
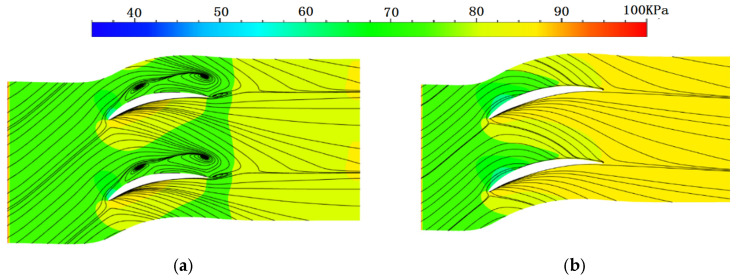
Endwall static pressure and wall limit streamline of cascade. (**a**) Straight cascade. (**b**) Bowed LESB cascade.

Figure 12 shows the endwall static pressure and wall limit streamline of the straight cascade and bowed LESB cascade (the other four near-endwall modeling methods have a similar flow field as that of the bowed LESB cascade). In the figure, there are two vortexes in the passage near the endwall of the straight cascade, and both vortexes are generated on the suction side. A small vortex can also be found at the trailing edge of the cascade. In the meantime, the five optimal cascades all improve the flow behavior greatly near the endwall, completely eliminating the vortex in the cascade passage and simplifying the flow form.

**Figure 13:**
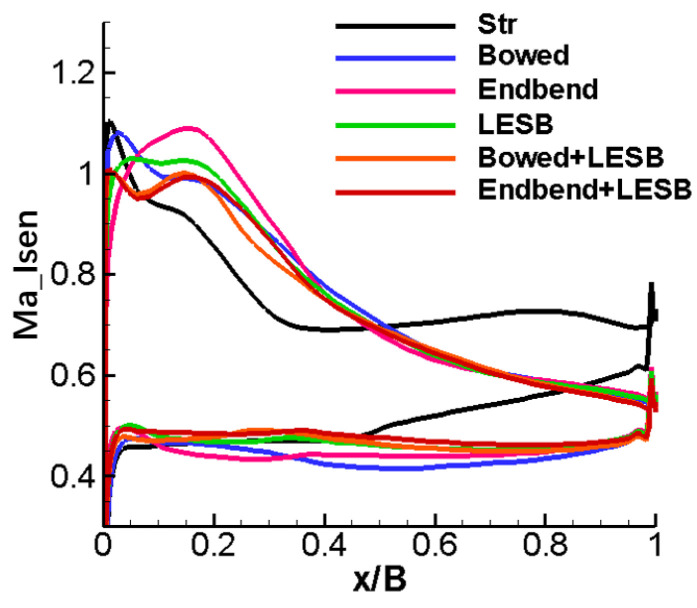
Distribution of isentropic Mach number on the blade surface near the endwall.

Figure 13 shows the distribution of the isentropic Mach number on the blade surface near the endwall. From the figure, the five endwall modeling cascades have approximately similar load distributions: much greater than the straight cascade before the first 50% relative chord length and almost the same on the rear 50% relative chord length. So, on the whole, the five endwall modeling cascades have a higher aerodynamic load than the straight cascade.

Preceding parts of the text found that many vortexes exist in the passage of the straight cascade. In order to make the vortex structure observable, we can observe the spatial streamline diagram. In the spatial streamline of the straight cascade and optimal cascade, we can find that the optimal cascades have effectively decreased the strength of the passage vortex and improved the flow condition at the endwall area. Comparing the five optimal cascades, the optimal bowed cascade and optimal bowed LESB cascade have the most significant radial migrations of the endwall boundary layer. In other words, they move more low-energy fluids from the endwall area to the mid-span area of the cascade, which can improve the flow condition near the endwall area and cause a detrimental effect at the middle section of the cascade. This phenomenon can also be observed in Figure 11. It can be seen from the figure that the streamlines flowing from the hub and the shroud will flow toward the mid-span area and eventually merge into the separation line. The strength of radial migration can be roughly measured by the height at the location where the streamlines merge into the separation lines. From the heights labeled in Figure 11 as H_RM, it can be found that the optimal bowed cascade and the optimal bowed LESB cascade have the greatest heights for radial migration of the endwall boundary layer, which is consistent with what was observed in the spatial streamline diagram.

**Figure 14:**
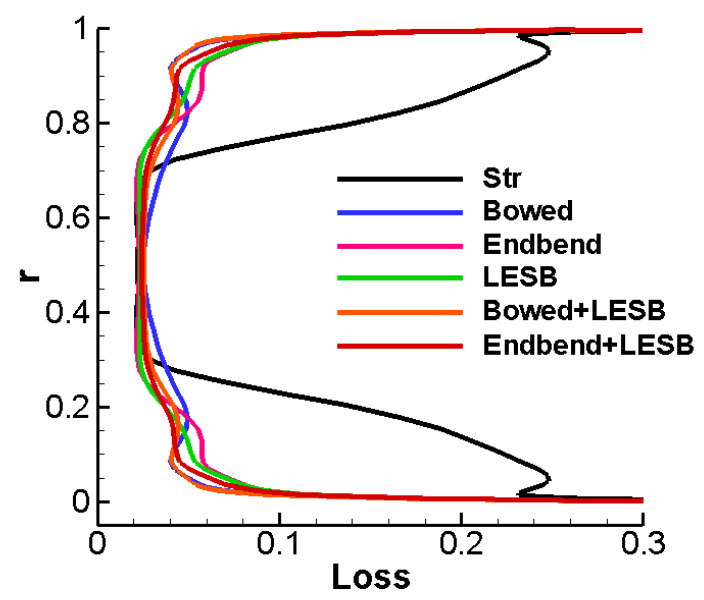
The radial distribution of cascade total pressure loss.

Figure 14 shows the radial distribution of the cascade total pressure loss. In the figure, we can find that the five optimized cascades have apparently smaller loss than the straight cascade in the area below 30% leaf height. In the region below 10% blade height, the optimal bowed cascade and optimal bowed LESB cascade have the smallest loss scheme, and they also have the biggest loss in the mid-span area at the same time. However, the bowed LESB cascade has the best performance near the endwall area with a little larger performance lose in the mid-span area. So, putting it all together, the bowed LESB cascade possesses the best aerodynamic performance.

### 5.3. Optimization Results of the Cascade Off-Design Performance

**Figure 15:**
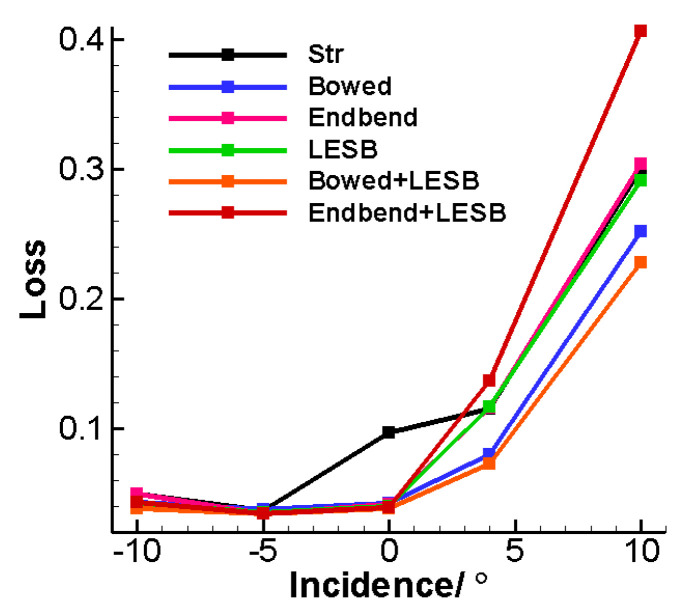
Cascade characteristic curves of the relationship between total pressure loss and incidence.

Figure 15 shows the cascade characteristic curves of the relationship between total pressure loss and incidence. It can be found that, compared with the straight cascade, the five optimal cascades have significantly less losses under the design conditions, but this is not the case when increasing the incidence. The endbend cascade, the LESB cascade and the endbend LESB cascade all have roughly the same total pressure loss as the straight cascade at 4° incidence, and the optimal bowed LESB cascade has the smallest loss magnitude at this incidence condition. However, when increasing the incidence to 10°, the loss of the optimal endbend LESB cascade is much larger than that of the other cascades. At this incidence, the optimal endbend cascade and the optimal LESB cascade both have a similar loss to the straight cascade, and the optimal bowed cascade and the optimal bowed LESB cascade have the minimum loss. At −5° incidence, all six schemes have a similar loss magnitude, as well as with the −10° incidence, though the optimal bowed LESB cascade is slightly better than others. In general, the bowed LESB cascade has the best aerodynamic performance under all incidence conditions. The endbend LESB cascade performs well under the design and negative incidence conditions, but a significant reduction of its performance can be observed under the positive incidence, even worse than straight cascade. The performance of the other three optimal cascades is good under the design incidence condition, but it is not ideal under off-design conditions.

### 5.4. Analysis on the Influence of Modeling Parameters of Bowed LESB Cascade

**Figure 16:**
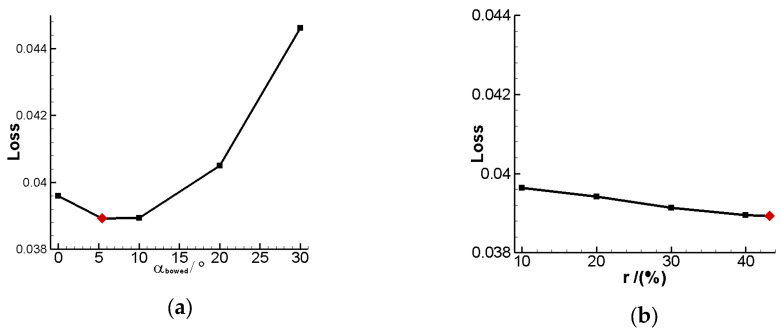

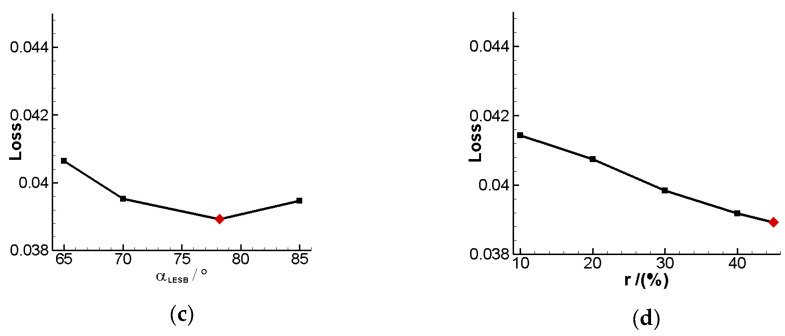
Total pressure loss variation of the optimal bowed LESB cascade with change of modeling parameters. (**a**) Loss—α_Bow. (**b**) Loss—H_Bow. (**c**) Loss—α_LESB. (**b**) Loss—H_LESB.

This study proposes two approaches for shaping the cascade at the endwall area, namely the bowed LESB cascade and endbend LESB cascade. Among all endwall modeling methods, the bowed LESB cascade has the best aerodynamic performance in this study. In order to comprehensively understand this modeling method, this section further investigates the effect of various parameters in endwall modeling on its aerodynamic performance. Figure 16 gives the total pressure loss of the optimal bowed LESB cascade that varies with modeling parameters. From the Figure 16a, it can be seen that the total pressure loss shows a trend of first decreasing and then increasing with the increasing of α_Bow, and it reaches minimum total pressure loss when α_Bow is 5.45°. From Figure 16b, we can find that the loss of the cascade keeps going down as H_Bow increases, and it reaches minimum loss at 45% blade height. However, compared with the angle, the effect of the camber height on cascade aerodynamic performance is relatively weak. From Figure 16c, it can be seen that the total pressure loss has a similar trend as Figure 16a, and the best performance point appears when α_LESB equals 78.21°. Figure 16d gives the effect of H_LESB; in the figure, we can find that the total pressure keeps going down while H_LESB keeps going up, just like in Figure 16b. Meanwhile, α_LESB and H_LESB both have similar influence relevance toward cascade aerodynamic performance. Based on the above analysis and research, it can be verified that the cascade optimized in this study has the best aerodynamic performance in all the bowed LESB cascades.

## 6. Conclusions

This paper has studied the optimization design method of high-loaded cascades considering the endwall effect and proposed two new near-endwall modeling methods: the bowed LESB method and endbend LESB method. At the same time, the modeling method of the LESB blade has been improved so that the generated airfoil has better continuity. This paper developed a high-loaded cascade optimization design platform containing five near-endwall blade modeling methods, and it is capable of coupled optimization toward airfoil and near-endwall modeling parameters. Based on this optimization design platform, a low solidity optimization design of the compressor stator cascade was carried out. The solidity of the optimized cascade decreases from 1.53 to 1.25 while the total pressure loss is only 0.0389, and the diffusion factor reaches roughly 0.6. In this study, five different near-endwall blade modeling methods, i.e., bowed blade, endbend blade, LESB blade, bowed LESB blade and endbend LESB blade, are used to conduct a full three-dimensional optimization design study on a high-loaded cascade. The following conclusions can be drawn:

(1) Synthetically comparing five different optimal near-endwall modeling cascades among design and off-design conditions, they all could improve the performance for the original cascade, while the optimal bowed LESB cascade possesses the minimum total pressure loss and maximum aerodynamic load at the same time. Except for the optimal bowed cascade, the aerodynamic performance of the other four optimal cascades is highly consistent. For the endbend blade, the positive endbend has better performance, and for the endbend LESB blade, the negative endbend has better performance.

(2) Comparing the flow field of the five optimal cascades, the optimal bowed cascade is the one wherein the axial position of the separation line is the nearest to the blade leading edge, while the other four optimal cascades’ axial positions of the separation line are similar. The separation line in the optimal bowed LESB cascade has the narrowest radial influence range.

(3) The straight cascade will form intense passage vortexes in the cascade passage, which will deteriorate the aerodynamic performance. After the near-endwall optimization design, the structure of the channel vortex was completely changed and weakened a lot.

(4) The radial migration of the low-energy fluid in the endwall boundary layer will have a great impact on the performance of the cascade. Among the five different optimal cascades, the optimal bowed cascade and the optimal bowed LESB cascade had the largest radial migration height of the low-energy fluid in the endwall boundary layer, so the loss of the two at the near-endwall area is the smallest.

(5) For the bowed LESB cascade, the bowed angle has the most significant influence on the cascade aerodynamic performance, while the bowed height has the weakest influence. Meanwhile, the leading-edge strake angle and the leading-edge strake height have almost the same influence strength on its performance.

## Figures and Tables

**Table 1:** Highly loaded cascade calculation results in different endwall mesh scales.

Mesh Scale at Wall/m	$1\;\times \;10^{-4}$	$5\;\times \;10^{-5}$	$1\;\times \;10^{-5}$	$5\;\times \;10^{-6}$	$1\;\times \;10^{-6}$	$5\;\times \;10^{-7}$
Total pressure loss	0.0416	0.0659	0.0968	0.0965	0.0948	0.0957
Static pressure ratio	1.1897	1.1694	1.1467	1.1472	1.1478	1.1477

**Table 2:** Cascade main design parameters.

Variable Name	Value
Blade inlet angle/°	42.0
Blade outlet angle/°	−8.0
Blade axial chord/mm	65
Aspect ratio	2.77
Solidity	1.25
Inlet Mach number	0.7
Incidence/°	0

**Table 3:** Specific parameters for five near-endwall modeling methods.

Near-Endwall Modeling Method	Parameters
Bowed Cascade	H_Bow, α_Bow
Endbend Cascade	H_G, α_G
Lesb Cascade	H_LESB, α_LESB
Bowed Lesb Cascade	H_Bow, α_Bow, H_LESB, α_LESB
Endbend Lesb Cascade	H_G, α_G, H_LESB, α_LESB

**Table 4:** Disturbance range of optimization variable.

Variable	Lower Bound	Upper Boundary
BFB	0.2	0.8
FS	0.2	0.8
XLMB	0.3	0.7
r1/mm	0.1	1.5
H_Bow	1%	45%
α_Bow/°	0	75
H_G	1%	45%
α_G/°	−10	10
H_LESB	1%	45%
α_LESB/°	10	90

**Table 5:** Parameter settings of improved artificial bee colony algorithm.

Variable	Colony Size	Max Cycle	Limit	r
Value	100	100	100	1.0

**Table 6:** Results of ultimate optimization variables.

Optimization Variable	Straight Cascade	Bowed Cascade	Endbend Cascade	LESB Cascade	Bowed LESB Cascade	Endbend LESB Cascade
BFB	0.363	0.337	0.367	0.371	0.365	0.360
FS	0.500	0.612	0.347	0.299	0.227	0.325
XLMB	0.658	0.674	0.496	0.422	0.349	0.470
r1/mm	0.611	0.784	0.879	0.914	0.699	0.762
H_Bow	-	38.15%	-	-	43.19%	-
α_Bow/°	-	16.60	-	-	5.45	-
H_G	-	-	11.73%	-	-	7.65%
α_G/°	-	-	+7.33	-	-	−5
H_LESB	-	-	-	45%	45%	45%
α_LESB/°	-	-	-	82.74	78.21	75.09%

**Table 7:** Optimization design result of high-loaded cascade.

Optimization Result	Total Pressure Loss	Diffusion Factor	Static Pressure Rise
Straight cascade	0.0965	0.5489	1.1472
Bowed cascade	0.0425	0.5889	1.1946
Endbend cascade	0.0416	0.5938	1.1951
LESB cascade	0.0402	0.5947	1.196
Bowed LESB cascade	0.0389	0.5957	1.1971
Endbend LESB cascade	0.0393	0.5956	1.1969
